# 磁固相萃取-液相色谱-质谱测定环境水样中全氟化合物及风险评估

**DOI:** 10.3724/SP.J.1123.2024.08015

**Published:** 2025-06-08

**Authors:** Chenshu GU, Zhenzhen LIU, Huiling JIN, Xiaoqi LIU, Meiyu WANG, Weijie SUN, Yangying SUN, Peipei QI

**Affiliations:** 1.宁波大学食品科学与工程学院，省部共建农产品质量安全危害因子与风险防控国家重点实验室，浙江 宁波 315211; 1. State Key Laboratory for Managing Biotic and Chemical Threats to the Quality and Safety of Agro-products，School of Food Science and Engineering，Ningbo University，Ningbo 315211 China; 2.浙江省农业科学院农产品质量安全与营养研究所，省部共建农产品质量安全危害因子与风险防控国家重点实验室，浙江 杭州 310021; 2. State Key Laboratory for Managing Biotic and Chemical Threats to the Quality and Safety of Agro-products，Institute of Agro-product Safety and Nutrition，Zhejiang Academy of Agricultural Sciences，Hangzhou 310021，China; 3.嘉兴市农业科学研究院桐乡农业科学研究所，浙江 嘉兴 314500; 3. Tongxiang Institute of Agriculture Science，Jiaxing Academy of Agriculture Science，Tongxiang 314500，China

**Keywords:** 磁固相萃取, 超高效液相色谱-串联质谱, 全氟化合物, 水, magnetic solid-phase extraction （MSPE）, ultra-high performance liquid chromatography-tandem mass spectrometry （UHPLC-MS/MS）, perfluorinated compounds （PFCs）, water

## Abstract

全氟及多氟烷基化合物（PFCs）在全球环境水体中广泛存在。据报道，饮用水的摄入可能是人类接触PFCs的重要来源，因此亟需针对饮用水中PFCs的赋存特征、来源分析和风险评估进行深入的探究。本文以磁性聚苯乙烯-吡咯烷酮（Fe_3_O_4_-PLS）为磁性吸附剂，建立了水中11种PFCs富集的磁固相萃取方法，结合液相色谱-串联质谱（LC-MS/MS）分析，实现了水中PFCs的准确、灵敏测定。Fe_3_O_4_-PLS经甲醇活化后置于水溶液中，超声辅助萃取15 min，经磁分离后，采用乙腈（含0.1%甲酸）将目标物洗脱，洗脱液经氮吹复溶后进样分析。方法验证结果表明，11种PFCs在1～100 μg/L范围内线性关系良好，相关系数（*R* ^2^）为0.997 6~0.999 9；检出限为0.001～0.620 ng/L，定量限为0.002～2.065 ng/L，方法灵敏度高。11种PFCs在不同添加水平（0.05、1、10、50 μg/L）下，回收率为60.8%～120.0%，相对标准偏差（RSD）为1.0%～20.0%，可满足水中PFCs的分析需求。采用该方法分析了杭州东苕溪15个点位（临近工厂、水库、居民区）样品中11种PFCs的残留浓度，共检测到6种PFCs，分别是全氟辛酸（PFOA）、全氟壬酸（PFNA）、全氟辛基磺酸（PFOS）、全氟庚基磺酸（PFHpS）、全氟丁基磺酸（PFBS）和全氟癸酸（PFDA），检出质量浓度为11.4~30.7 ng/L，其中全氟辛酸的质量浓度最高达到25.5 ng/L。东苕溪中PFCs主要来源于前体降解和工业废水排放，流域地表水中PFCs远低于官方给出的健康参考值，尚未达到对生态和人体产生风险的水平。

## Abstract (en)

Perfluorinated compounds （PFCs） are widely used， persistent， and their presence in water is of significant concern. PFCs， particularly short-chain variants， are highly soluble and mobile in water， which enables their transport over long distances via river systems， potentially leading to extensive contamination. These compounds are resistant to degradation， which is challenging for conventional water-treatment methods that often remove PFCs ineffectively， leading to their prolonged presence in water bodies. This paper establishes a magnetic solid-phase extraction method for 11 PFCs in enriched water using magnetic polystyrene pyrrolidone （Fe_3_O_4_-PLS） as a magnetic adsorbent. Purified lipophilic PLS magnetic beads were used as the solid-phase extractant， and their surfaces were modified using phenyl and pyrrolidone groups to facilitate the adsorption of PFCs that contain hydrophilic functional groups and hydrophobic alkyl side chains. PFCs in water were determined accurately and sensitively by liquid chromatography-tandem mass spectrometry （LC-MS/MS）. The method involved accurately weighing 50 mg （±0.05 mg） of the Fe_3_O_4_-PLS into a 500-mL beaker， adding 2 mL of methanol （for activation）， placing the beaker on a magnet for 30 s， and discarding the methanol once the methanol and Fe_3_O_4_-PLS had been completely separated. A 200-mL aliquot of an aqueous mixed PFC solution was added to the beaker， sonicated for 15 min， and then placed on a strong magnet until the Fe_3_O_4_-PLS had completely separated at the bottom of the beaker. The upper liquid was discarded. A 4-mL acetonitrile containing 0.1% formic acid was added as the Fe_3_O_4_-PLS eluent， ultrasonicated for 30 s， after which the beaker was placed on the magnet and the eluate collected. The sample was taken to dryness under a stream of nitrogen， the residue was redissolved in 0.5 mL of acetonitrile， ultrasonicated for 10 s， and then membrane-filtered prior to analysis by LC-MS/MS. The 11 PFCs exhibited good linear relationships ranging from 1 to100 μg/L， with correlation coefficients （*R* ^2^） ranging from 0.997 6 to 0.999 9. Limits of detection and quantification （LODs and LOQs， respectively） were determined to be 0.001–0.620 ng/L and 0.002–2.065 ng/L， respectively， indicative of high sensitivity. The 11 PFCs exhibited recoveries of 60.8%–120.0% at various concentrations （0.05， 1， 10， and 50 μg/L）. Relative standard deviations （RSDs） ranged from 1.0% to 20.0%， which meet the requirements for PFC analysis in water. The concentrations of the 11 PFCs at 15 sites in the Dongtiaoxi River， Hangzhou （near factories， reservoirs and residential areas） were analyzed using the developed method. A total of six PFCs， namely perfluorooctanoic acid （PFOA）， perfluorononanoic acid （PFNA）， perfluorooctane sulfonic acid （PFOS）， perfluoroheptanoic acid （PFHpS）， perfluorobutanesulfonate （PFBS） and perfluorodecanoic acid （PFDA）， were detected at mass concentrations of 11.4–30.7 ng/L. The highest mass concentration of PFOA was determined to be 25.5 ng/L. The PFCs in the Dongtiaoxi River were found to be mainly associated with precursor degradation and industrial wastewater discharge. A sudden rise in the pollution level was observed near the sampling point with the lowest detected concentration， which is possibly ascribable to the strong hydrodynamic difference between the hydraulic transport process near the river wharf and the highly turbulent flowing water. This difference results in the suspended particles in surface water mixing with re-suspended sediment particles such that PFCs are released back into the surface water. Pollution levels were observed to continuously decrease in the directions of the extended river and its estuary and tributaries. Risk assessments revealed that the PFC levels in the surface water of the Dongtiaoxi River basin are much lower than the official health reference value and have not reached levels that are expected to cause ecological harm and risk human health. The data are expected to support monitoring-system improvements by providing an in-depth understanding of occurrence characteristics， and help formulate a management plan for PFCs in the Dongtiaoxi River.

全氟化合物（perfluorinated compounds，PFCs）是碳链上的氢原子被氟原子取代所形成的含共价键的有机化合物^［1］^，因其高化学稳定性、热稳定性和优良的疏水疏油性在工业及商业生产领域备受青睐，广泛应用于纺织品、皮革制品、食品添加剂、高分子助剂等领域。但PFCs的C-F键呈强极性，在臭氧、微生物作用和氧化还原作用下难以完全降解，导致PFCs易在水生环境中富集并通过食物链迁移放大向上级生物体甚至人体进行转移累积，从而导致内分泌干扰、生殖毒性和致畸致癌性等毒性作用。

目前PFCs已在环境水体中广泛检出，包括上海黄浦江^［2］^、黄海^［3］^、太湖^［4］^等。关于浙江省地表水PFCs污染状况的研究主要集中在钱塘江-大运河水系^［5］^，包括西湖、太湖、嘉兴段和杭州段的大运河。苕溪是浙江省八大水系之一，属于太湖流域的主要支流。杭州段的苕溪流域存在5种不同的土地利用方式，分别为农田、林地、居民区、工业区和城区^［6］^，东苕溪主要以林地和耕地为主，其次是城镇用地等。东苕溪不仅承担着供水职能，还兼顾着排污、运输、灌溉等其他职能^［7］^。《浙江省水功能区、水环境功能区划分方案》划分东苕溪流域为杭州市民饮用水水源保护区，以地表水质量标准中Ⅲ类水质标准进行控制。根据东苕溪中下游的21项常规检测指标，发现其五日生化需氧量超过Ⅲ类水质标准，说明存在较高的有机污染，已经构成了对东苕溪水源地水质安全的潜在威胁。然而，现有关于东苕溪的研究中主要关注氮磷水平，对新型有机污染物水平的评价较少。因此，检测苕溪水体中PFCs残留浓度，摸清本底水平，对水体中新污染物控制具有重要的意义。

PFCs分析多采用液相色谱-串联质谱（LC-MS/MS），由于环境水体中污染物赋存浓度普遍比较低，分析水体中未知的目标物时需要采集大体积的水样。所以在进行LC/MS-MS分析之前需要对样品进行富集浓缩净化等前处理。目前，环境水样中PFCs的前处理技术主要有溶剂萃取、固相萃取（SPE）、分散固相萃取（d-SPE）和磁固相萃取（magnetic solid-phase extraction，MSPE）。江桂斌研究团队利用亲水-疏水平衡（HLB）固相萃取柱^［8］^、PWAX固相萃取柱^［9］^以及WAX固相萃取柱^［10］^等不同的SPE柱，成功实现了河水、自来水等样品中多种PFCs及其新型替代物质的准确分析。SPE技术富集、净化能力强，然而溶剂消耗较大，操作过程复杂，耗时长。MSPE技术是SPE的延伸，它以磁性材料为分散吸附剂，磁吸附剂可以完全分散于环境水样中，在短时间内吸附和萃取目标物，并可以在外磁场下快速分离和收集^［11］^，具备易于操作、萃取时间短、样品用量少、有机溶剂用量低和环境友好等突出优势。磁性吸附剂的选择对MSPE的萃取效率、富集因子、选择性和抗干扰能力有着显著影响，是实现高效萃取的关键。据报道，以二乙烯基苯和吡咯烷酮为功能单体在磁球表面修饰获得的两亲型磁聚合物（Fe_3_O_4_-PLS）具有良好的水相分散性和吸附性能，并应用于近百种农药残留的分析测定^［12‒14］^。故而，考虑到羧酸类和磺酸类PFCs均具有亲水和疏水功能端基，预期以Fe_3_O_4_-PLS为吸附剂可获得较好的富集性能。

基于此，本研究拟以Fe_3_O_4_-PLS为磁吸附剂，验证MSPE-LC-MS/MS方法分析水体中PFCs的可行性和方法性能。采集东苕溪下游区域典型水样品，分析了不同位点东苕溪地表水中PFCs的组成、浓度水平和残留状况，明确其赋存特征，并利用风险熵法开展风险评估研究，为东苕溪中PFCs的污染评估提供了数据支撑。

## 1 实验部分

### 1.1 仪器、试剂与材料

Nexera X2超高效液相色谱仪、8050三重四极杆质谱仪和Labsolution分析软件（日本Shimadzu公司）。Filter Unit滤膜（0.22 μm，天津博纳艾杰尔科技有限公司）。超声波清洗仪（昆山市超声仪器有限公司）。

甲醇、乙腈（色谱纯）购于美国Merck公司；甲酸（分析纯）、乙酸铵（分析纯）购于美国Tedia公司。实验用水为超纯水（Milli-Q超纯水系统制备，美国Millipore公司）。Fe_3_O_4_-PLS为实验室自制，粒径约760 nm，制备方法参照文献［^15^］。11种PFCs标准品（95%~99%）均购于天津阿尔塔科技有限公司。

### 1.2 实验方法

#### 1.2.1 样品前处理

准确称取50 mg（±0.05 mg）Fe_3_O_4_-PLS于500 mL烧杯中，加入2 mL甲醇匀速摇晃30 s，以确保甲醇与Fe_3_O_4_-PLS完全活化，后将烧杯置于超强磁铁上静置30 s，待甲醇和Fe_3_O_4_-PLS完全分离后，弃去甲醇。将200 mL PFCs混合水溶液加入装有被活化Fe_3_O_4_-PLS的烧杯中，超声15 min，后将烧杯静置于超强磁铁1 min，直至Fe_3_O_4_-PLS完全吸附在烧杯底部，弃去上层液体，向烧杯中加入4 mL 0.1%甲酸乙腈溶液，超声30 s对Fe_3_O_4_-PLS进行洗脱，后将烧杯置于磁铁上，收集洗脱液，随后氮吹至近干，加入0.5 mL乙腈复溶，超声10 s后过滤膜，待LC-MS/MS分析。

#### 1.2.2 仪器分析条件

Luna Omega C18色谱柱（100 mm×2.1 mm，1.6 μm，美国Phenomenex公司）；柱温为35 ℃；进样体积为2 μL；流动相A为含10 mmol/L乙酸铵的甲醇-水（80∶20，v/v）溶液，B相为含10 mmol/L乙酸铵的甲醇溶液；梯度洗脱程序：0~10 min，10%B~100%B；10~14 min，100%B；14~14.5 min，100%B~10%B；14.5~15 min，10%B；流速0.25 mL/min。

质谱条件：采用电喷雾离子源（ESI），多反应监测模式；扫描方式：正离子扫描；毛细管电压：4000 V；毛细管温度：300 ℃；加热块温度：400 ℃；去溶剂化温度：250 ℃；氮气干燥气流速：10 L/min；空气加热气流速：10 L/min；氮气雾化气流速：3 L/min；氮气发生器；脱溶剂气流量：700 L/h；锥孔气流量：50 L/h；碰撞气：氩气。11种PFCs的母离子、子离子等参数见表1。

**表 1:** 11种目标PFCs的质谱参数

Compound	Abbreviation	Parent ion （*m/z*）	Product ions （*m/z*）	DPs/V	CEs/eV	CXPs/V
Perfluorobutanesulfonat	PFBS	298.8	79.9^*^； 99.0	-71.2； -70.2	-51.2； -40.0	-3.5；-5.1
Perfluorohexanesulfonate	PFHxS	398.8	79.9^*^； 99.0	-75.3； -72.6	-66.0； -51.4	-3.1；-2.0
Perfluoroheptanoic acid	PFHpS	448.8	79.9^*^； 99.0	-81.0； -85.0	-75.0； -56.1	-3.6；-2.0
Perfluorooctane sulfonic acid	PFOS	498.8	79.9^*^； 99.0	-89.1； -82.0	-86.1； -62.7	-3.4；-2.0
Perfluorodecane sulfonic acid	PFDS	599.3	79.9^*^； 99.0	-97.0； -98.2	-76.0； -81.0	-3.6；-2.0
Perfluorooctanoic acid	PFOA	412.8	369.0^*^； 168.8	-16.5； -20.6	-15.2； -24.5	-10.4；-4.4
Perfluorononanoic acid	PFNA	462.8	419.1	-31.4	-19.1	-12.1
Perfluorodecanoic acid	PFDA	512.8	469.1	-24.4	-24.0	-7.4
Perfluoroundecanoic acid	PFUnDA	562.8	519.1	-23.4	-30.0	-8.5
Perfluorolauric acid	PFDoDA	612.8	569.0	-24.0	-25.6	-9.2
Perfluorotetradecanoic acid	PFTeDA	712.8	669.0	-52.9	-21.1	-11.2

* Quantitative ion； DPs： declustering potentials； CEs： collision energies； CXPs： cell exit potentials.

#### 1.2.3 风险评估方法

采用风险熵值（risk quotients，RQ）来评价PFCs对水生生物的潜在影响。RQ定义为污染物实测的环境浓度（measured environmental concentration，MEC）与预测的无效应浓度（predicted no effect concentration，PNEC）的比值^［16］^，计算公式为RQ=MEC/PNEC。当RQ≥1.0时，认为污染物对水生生物存在高风险影响；当0.1≤RQ<1.0时，认为存在中等程度风险，当RQ<0.1时认为存在低风险。

采用健康风险值（hazard ratio，HR）来评价污染物的健康风险。HR定义为每日平均摄入量（average daily intake，ADI）与参考剂量（reference dose，RfD）的比值^［17］^，计算公式为HR=ADI/RfD，ADI=*ρ*×*V*/*m，*其中，RfD为参考剂量的参考值（ng/（kg·day）），ADI为每日平均摄入量（ng/（kg·day））， *ρ*是PFCs实测的质量浓度（均值，ng/L），*V*为日均饮水摄入量（均值，L/d）；*m*为人体质量（kg）。当HR>1.0时，认为污染物对人体健康存在风险；当0.1≤HR<1.0时，认为存在潜在风险；当HR＜0.1时，认为污染物对人体健康无风险。

## 2 结果与讨论

### 2.1 水中PFCs的MSPE-LC-MS/MS方法验证

#### 2.1.1 线性关系、灵敏度及基质效应评价

方法验证包括线性关系、基质效应、检出限（LOD）、定量限（LOQ）、回收率和精密度。分别以空白样品基质溶液和乙腈为溶剂制备基质匹配标准溶液和溶剂标准溶液，标准溶液质量浓度为0.01、0.02、0.05、0.1、0.5、1、5、10、50、100、200 μg/L。经LC-MS/MS分析后，通过绘制分析物的峰面积与质量浓度的线性关系拟合标准曲线和基质匹配标准曲线，得到回归方程和相关系数（*R* ^2^）。结果见表2，11种PFCs在1～100 μg/L范围内有良好的线性关系，*R* ^2^均大于0.997 5。以信噪比（*S/N*）为3和10分别确定LOD为0.000 2~0.248 μg/L，LOQ为0.000 6~0.826 μg/L；由于样品前处理过程中的浓缩倍数为400，所以LOD为0.001～0.620 ng/L，LOQ为0.002～2.065 ng/L。

**表 2:** 水体中11种PFCs回归方程、相关系数、基质效应、线性范围、检出限和定量限

Compound	Regression equation	*R* ^2^	Matrix effect	LOD/（ng/L）	LOQ/（ng/L）
PFBS	*Y*_1_=13277*X*+129032	0.9993	0.96	0.038	0.128
	*Y*_2_=13768*X*+6913.9	0.9998		0.020	0.073
PFHxS	*Y*_1_=5572.4*X*+1520	0.9999	1.08	0.620	2.065
	*Y*_2_=5156.7*X*+5269.3	0.9997		0.048	0.158
PFHpS	*Y*_1_=120152*X*+65355	0.9999	0.98	0.078	0.255
	*Y*_2_=119289*X*+17287	0.9999		0.048	0.155
PFOS	*Y*_1_^=^40589*X*+50713	0.9996	1.02	0.065	0.213
	*Y*_2_=40467*X*+25291	0.9997		0.048	0.160
PFDS	*Y*_1_=113789*X‒*3115.4	0.9998	1.06	0.055	0.185
	*Y*_2_=97174*X*+53300	0.9998		0.028	0.093
PFOA	*Y*_1_=4136.6*X*+983319	0.9976	1.58	0.001	0.002
	*Y*_2_=2610.8*X*+548998	0.9999		0.001	0.003
PFNA	*Y*_1_=38178*X*+66566	0.9990	1.55	0.027	0.096
	*Y*_2_=24540*X*+6410.3	0.9998		0.003	0.080
PFDA	*Y*_1_=51475*X*+38877	0.9995	1.72	0.005	0.013
	*Y*_2_=29820*X*+16793	0.9999		0.013	0.043
PFUnDA	*Y*_1_=35117*X*+60230	0.9990	1.56	0.008	0.030
	*Y*_2_^=^22463*X*+29509	0.9978		0.008	0.026
PFDoDA	*Y*_1_=2523.8*X‒*26116	0.9998	1.06	0.018	0.058
	*Y*_2_=2360.8*X‒*1946.3	0.9999		0.038	0.125
PFTeDA	*Y*_1_=13131*X*+19480	0.9985	1.03	0.108	0.365
	*Y*_2_=18079*X‒*543.6	0.9998		0.020	0.070

*Y*： peak area of target analyte （*Y*_1_： matrix standard； *Y*_2_： solvent standard）； *X*： mass concentration of target analyte， μg/L.

基质效应（matrix effect，ME）通过基质匹配标准溶液曲线的斜率和溶剂标准溶液曲线的斜率之比进行确定，如果斜率比为1，说明基质效应为0；如果斜率比小于1，为基质抑制效应；如果斜率比大于1，为基质增强效应^［18］^。结果见表2，11种PFCs的基质效应在0.2～2.0，说明存在不同程度的基质抑制或增强效应，其中63.3%的目标物ME值在0.9~1.1之间，说明基质效应对这些化合物的定量分析结果影响程度较低，而PFNA（1.55）、PFOA（1.58）、PFUnDA（1.56）、PFDA（1.72）的ME值高于1.5，说明存在明显的基质增强效应，需要采用基质匹配标准曲线进行校正才可消除基质影响，得到准确的定量分析结果。

#### 2.1.2 准确度和精密度

按照样品前处理方法，在空白纯水样品中添加低、中、高3个水平（0.05、1、10、50 μg/L）的标准品，采用LC-MS/MS测定，计算出不同水平下的回收率，精密度以计算得出的相对标准偏差（RSD）进行确定。回收率结果见表3，可以看出，11种PFCs在不同添加水平下的平均回收率为60.8%～120.0%，RSD低于20%。

**表 3:** 水体中不同水平下11种PFCs的回收率和RSD（*n*=5）

Compound	Recoveries （RSDs）/ %			
	0.05 μg/L	1 μg/L	10 μg/L	50 μg/L
PFBS	77.8 （7.8）	83.4 （3.9）	89.4 （10.0）	92.3 （2.4）
PFHxS	82.9 （18.5）	88.1 （4.2）	85.7 （12.7）	62.8 （4.1）
PFHpS	82.5 （2.2）	89.3 （14.5）	76.1 （11.8）	78.5 （5.9）
PFOS	89.6 （5.7）	89.3 （13.3）	85.9 （6.6）	80.4 （6.5）
PFDS	76.2 （3.7）	90.6 （3.5）	85.1 （10.8）	81.8 （9.8）
PFOA	99.3 （6.0）	63.6 （14.1）	113.5 （5.5）	106.4 （13.2）
PFNA	119.4 （16.8）	94.4 （13.2）	115.3 （7.0）	114.3 （6.3）
PFDA	96.2 （0.9）	91.1 （14.9）	113.6 （13.8）	120.0 （5.0）
PFUnDA	74.6 （4.2）	118.3 （12.3）	116.9 （13.9）	120.0 （7.6）
PFDoDA	95.1 （4.6）	119.4 （14.3）	110.3 （12.7）	84.7 （8.5）
PFTeDA	89.8 （8.5）	103.1 （5.5）	100.4 （6.3）	90.8 （6.9）

### 2.2 东苕溪地表水中PFCs的赋存特征与风险评估

为全面调查东苕溪流域地表水中PFCs的赋存水平，我们根据东苕溪水系的实际情况，在中国浙江省杭州市东苕溪河水内共采集15个表层水样（0～10 cm），其中东苕溪主干流按水流方向依次为G1~G11，在主干G11处有两条支流，支流1的分流处和末端处分别设置为Z1和Z2，在支流的2的分流处和末端处分别设置为Z3和Z4。采样前用纯水清洗采样器、冷藏箱及存放水样的聚乙烯瓶，每个样品容量为500 mL；为防水样污染，采集后将水样置于冷藏箱保存，每个地点的实际样品设置3个平行组。

#### 2.2.1 地表水中PFCs的浓度水平与组成特征

东苕溪地表水中共检出 6种PFCs，∑PFCs的含量范围为11.4～30.7 ng/L，其中PFOA检出含量最高，为7.9～25.5 ng/L，其次是PFOS和PFNA。表4对比了国内外部分地表水中PFOS和PFOA的检出水平，结果表明PFOA在东苕溪地表水中的检出水平低于黄浦江、西溪湿地和汉江，略高于青山水库、千岛湖的地表水，普遍低于韩国洛东江、日本东京湾和新加坡冷岳河，属于相对清洁的程度。随着对于PFASs生物累积性研究的深入，目前普遍认为碳链长度大于7的PFCAs具有较为显著的生物富集效应^［19］^，根据图1显示，本次东苕溪地表水中检测到的全部为中短链PFCs。这可能与工业生产量和化合物性质相关，在工业生产上中短链PFCs以替代品的形式大规模地在原长链PFASs应用的各个领域得以推广和使用；其次，相较于长链的PFCs，中短链的PFCs具有较高的水溶性和解离度，更倾向于分配到水相而不是沉积物和悬浮颗粒物中。

**表 4:** 国内外地表水中PFCs赋存水平

Sampling location	Mass concentrations/（ng/L）			Ref.
	PFOS	PFOA	∑PFCs	
Qiandao Lake， China	ND	0.52-3.6	1.70-6.2	［^20^］
Caohai Lake， China	0.04-0.6	0.71-3.1	3.17-16.3	［^21^］
Xixi Wetland， China	ND	34.66-197.8	0.44-197.8	［^22^］
Huangpu River， China	<286	1.0-402.7	39.8-576.2	［^2^］
Pearl River， China	0.90-99	0.85-13.0	/	［^23^］
Luoma Lake， China	3.6-394	7.6-81.3	46.09-120.3	［^24^］
Tokyo Bay Basin， Japan	0.11-99.4	0.08-3704	1.57-6024.9	［^25^］
Red River， Vietnam	<0.03-0.04	0.1-0.4	0.23-1.5	［^26^］
Nakdong River， Korea	2.5-66	8.9-28.0	42-160	［^27^］
Marina catchment， Singapore	1.3-156	5.4-38.2	5.2-301.5	［^28^］
Dongtiaoxi River， China	2.40-4.5	7.88-25.8	9.87-29.9	this study

ND： not detected.

**图1:**
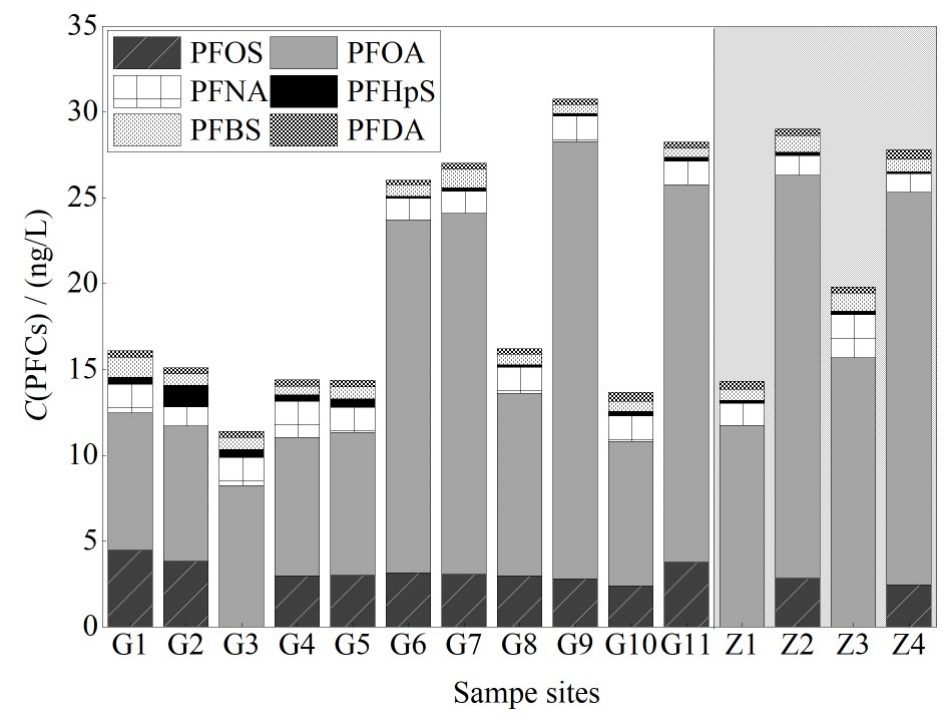
地表水样品中PFCs的质量浓度与组成

#### 2.2.2 东苕溪地表水中PFCs的分布特征

根据地表水中∑PFCs的检出情况（图1）可知，所有样品中检出的PFCs主要种类基本一致，表明污染物随水流而迁移。含量最高的采样点位分别在G9（30.7 ng/L）、G11（28.2 ng/L）和Z4（27.8 ng/L），调查发现其上游聚集着机械、建筑材料等生产企业，较高的PFCs含量可能是受到周围生产企业的影响。据研究报道，利用同系物比值评价可确定其潜在来源，PFOA/PFOS<1，则PFOS存在点源污染排放；PFOA/PFOS>1，说明PFOS存在降雨输入；当1<PFOA/PFOS<1.5时，则可能存在大气沉降产生的PFCs^［29］^。由图2可知，15个采样点PFOA/PFOS的比值均大于1，表明研究区域PFOS存在降雨输入。同时，由于PFHpA/PFOA的比值可以反映大气沉降对PFCs主要污染源的影响，而本研究中PFHpA在该流域具有较低的检出率，说明大气沉降并不是此地的污染来源。Paul等^［30］^提出环境中氟调聚物醇（FTOH）作为一种挥发性前体化合物可以降解形成PFNA、PFOA等，因此当PFOA/PFNA的比值在7～15时代表工业生产直接排放PFCs，当比值≥15时，意味着存在PFCs二次污染，即PFCs前体物氟调聚物醇的降解^［31］^。由图2可知，PFOA/PFNA在研究区域内的值为3.8～20.8 ng/L，G6、G7、G9、G11、Z2、Z4采样点的PFOA/PFNA均大于15，其中Z2、Z4大于20，表明这些地点中存在FTOH降解生成的PFCs，Z1采样点比值为7～15，说明存在直接的PFCs工业排放源。根据采样点信息，Z1、Z2、Z4靠近橡胶、机械和装饰电镀等化工行业的生产企业，这进一步证实了附近PFCs主要来源于前体降解和工业排放。

**图2:**
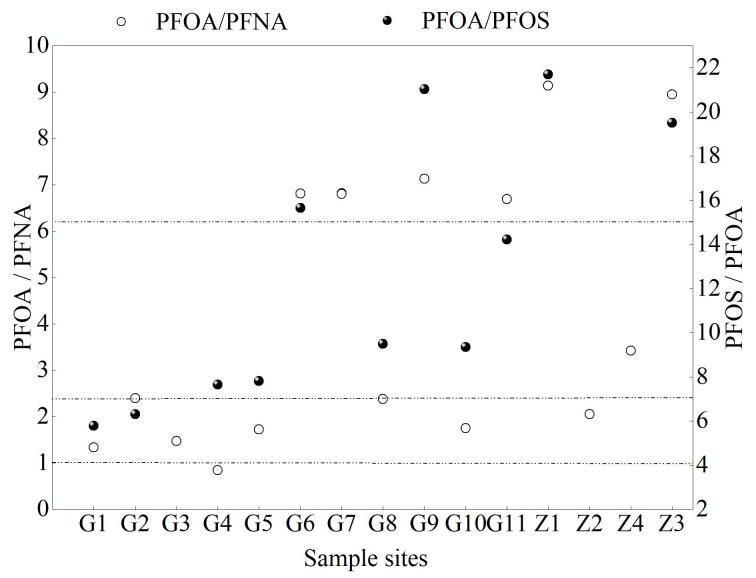
各采样点的PFOA/PFOS 、PFOA/PFNA值

检出浓度最低的采样点是G3，该采样点处于化工业密集区的上游，毗邻家庭农场、果蔬种植园区和水产养殖基地，污染源较少，同时临近的采样点（包括G5、G4、G2、G1）都呈现出相对低的污染水平。G7、G6点位虽然靠近低污染点G5，但污染水平骤然上升（总质量浓度分别为26.1 ng/L和27.1 ng/L），这可能是由于G7和G6临近河流码头，凹岸水位深，水力运输过程中的水动力差较强，水流的快速湍动使地表水中的悬浮颗粒与重新悬浮的沉积物颗粒混合^［32］^，使PFCs重新释放到表层水体，造成地表水污染水平的提高；G9和G11、Z2和Z4、Z1和Z3的污染水平呈现出沿河流-河口-支流方向连续降低的分布模式。

#### 2.2.3 地表水中PFCs的风险评估

根据2023年4月1日起施行的GB 5749-2022《生活饮用水卫生标准》对水质中PFCs的管控要求，PFOA和PFOS的限值分别是80 ng/L和40 ng/L，美国环境保护署（U.S. Environmental Protection Agency，EPA）规定的健康参考值则为200 ng/L和400 ng/L，可知东苕溪所有地表水中最具代表性的PFOA和PFOS浓度水平远低于官方给出的健康参考值。Yang等^［33］^参考美国环保局指南推荐的算法，计算出了PFOS和PFOA标准最高浓度（criterion maximum concentration，CMC）分别为3.8 mg/L和45.5 mg/L，标准连续浓度（criterion continuous concentration，CCC）为0.3 mg/L和3.5 mg/L；本研究中PFOA的实测质量浓度范围（7.9～25.5 ng/L）和PFOS的实测质量浓度范围（2.4～4.5 ng/L）远低于标准浓度值，表明东苕溪地表水中PFOS和PFOA对水生生物风险较低。

由于PFCs风险评估的参数相对匮乏，本文只针对赋存浓度较高的PFOA和PFOS分别进行生态和健康风险评价，应用风险熵法评价PFCs对水生生物和居民健康的风险影响，根据以往文献的计算和报道，评价阈值，PFOA和PFOS的PNEC 值分别取570 000 ng/L和1 000 ng/L^［34］^，在没有其他污染单体RfD值的情况下，PFCAs的RfD值假定与PFOA相似，PFSAs的RfD值假定与PFOS相似，PFOA和PFOS的RfD值为200和80 ng/（kg·day）^［17］^，依据《中国人群暴露参数手册（成人卷）》取日均饮水摄入量为1.85 L/d，居民平均体重为60.6 kg。如图3所示，东苕溪地表水PFOS的RQ值为PFOA的2～4倍，但二者的RQ值均小于0.1，表明该水域污染较为广泛的PFOA与PFOS尚未达到对水生生态环境具有风险的水平，潜在点源排放对水域影响不大，PFCs污染较轻，这与上述与其他水域PFCs污染情况进行对比所得出的结果一致。所有采样点PFCs的HR值均小于0.1，PFOA整体高于PFOS，表明该水域PFCs的污染浓度尚未达到对居民健康具有风险的水平。

**图3:**
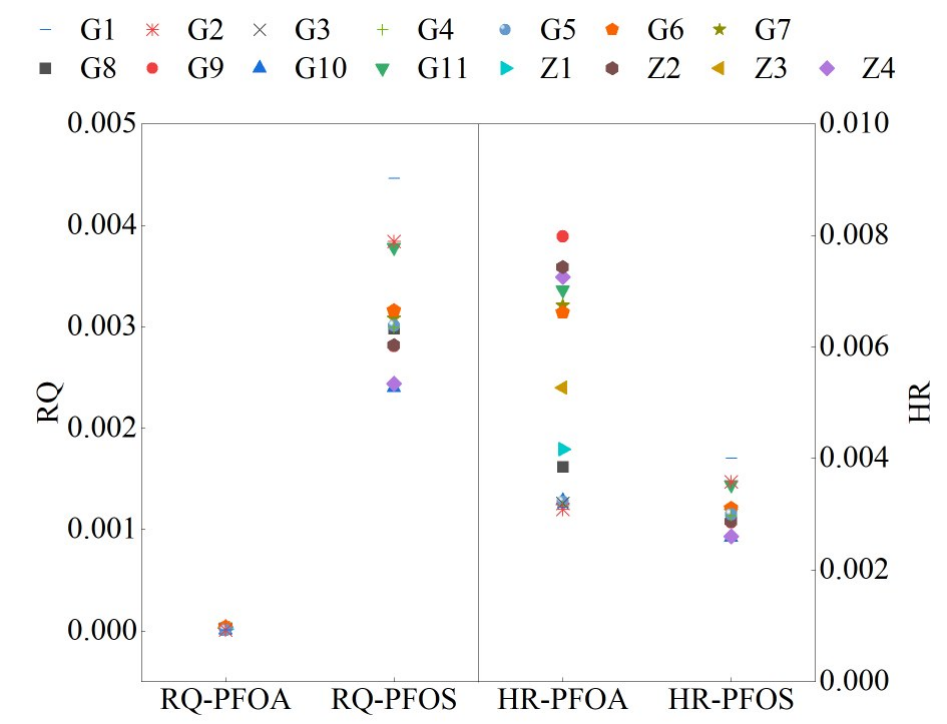
地表水中PFCs的风险值

## 3 结论

本研究成功建立了可满足环境水样中低浓度PFCs分析需求的基于Fe_3_O_4_-PLS的MSPE-LC-MS/MS方法，并进一步利用该方法对杭州东苕溪地表水中的PFCs赋存特征和风险评估进行了研究。本方法可广泛应用于河流、湖泊、地下水等各类水体中PFCs的检测，也可在工业废水处理、受污染水体修复等领域用于监测PFCs的去除效率，指导治理过程，确保饮用水的安全性，为制定相应的水质标准和管理措施提供科学依据。尽管本研究在PFCs检测方法的开发和应用方面取得了显著进展，但仍存在一些未能解决的问题：尽管采用了基质匹配标准曲线进行校正，但部分PFCs仍表现出明显的基质增强效应；此外，本方法的LOD和LOQ虽然已经达到了较高水平，但在某些极端低浓度环境水样中，仍可能存在检出限过高的问题。未来可以进一步深入研究PFCs的污染源，特别是前体化合物的降解机制和工业排放特征；开展多介质中PFCs污染的同步研究，分析PFCs在水-土-沉积物等不同介质中的分布特征、迁移转化规律以及相互作用机制，探究对水体污染的贡献；同时，开发高效的PFCs污染治理技术，如新型吸附剂、高级氧化技术等，提高吸附剂的吸附性能和选择性，以实现对PFCs污染的有效治理。

## References

[1] Huang J . ［MS Dissertation］. Harbin： Harbin Institute of Technology， 2011

[2] Sun Z， Zhang C， Yan H， et al . Chemosphere， 2017， 174： 127 28160676 10.1016/j.chemosphere.2017.01.122

[3] Wan Y， Wang S， Cao X， et al . Environ Monit Assess， 2017， 189（3）： 100 28185155 10.1007/s10661-017-5807-8

[4] Guo C， Zhang Y， Zhao X， et al . Chemosphere， 2015， 127： 201 25725312 10.1016/j.chemosphere.2015.01.053

[5] Peng B X， Hong W J， Li F F， et al . Environmental Chemistry， 2021， 40（10）： 3001

[6] Zhu C Y， Fu G W， Li G Y， et al . Sichuan Environment， 2021， 40（1）： 136

[7] Jin J， Yuan X Y， Chen S W， et al . Journal of Lake Sciences，2017， 29（3）： 594

[8] Lin Y F， Ding L， Ruan T， et al . Environmental Chemistry， 2015， 34（5）： 863

[9] Zhou H Y， Gong X J， Jiang J， et al . Textile Auxiliaries， 2024， 41（7）： 62

[10] Wei L E， Shao M H， Zhang J， et al . Acta Scientiae Circumstantiae， 2016， 36（5）： 172

[11] Li X L， Lü S Y， Zheng X N， et al . Journal of Nanjing Normal University （Engineering and Technology edition）， 2022， 22（2）： 87

[12] Tang X H， Song H L， Yao L Y， et al . Journal of China Pharmaceutical University， 2024， 55（4）： 485

[13] Zhang L， Li L， Zhang D Y， et al . Chinese Journal of Analysis Laboratory， 2024， 43（3）： 409

[14] Liu X， Teng J， Zhou Y， et al . Journal of Criminal Investigation Police University of China， 2022， 167（3）：111

[15] Liu Z， Qi P， Wang J， et al . Sci Total Environ， 2020， 708： 135221 31806340 10.1016/j.scitotenv.2019.135221

[16] Du G Y， Jiang X P， Zhuo L， et al . Ecology and Environmental Sciences， 2019， 28（11）： 2266

[17] Qiao X C， Zhao X R， Guo R， et al . Research of Environmental Sciences， 2019， 32（12）： 2148

[18] Yu M， Yao F， Zhang H， et al . Chinese Journal of Pesticide Science， 2022， 24（3）： 591

[19] Hao X W， Li L， Wang J， et al . Environmental Science， 2015， 36（8）： 3106 26592048

[20] Zhang M， Tang F L， Cheng X L， et al . Journal of Lake Sciences， 2020， 32（2）：337

[21] Zeng S Y， Yang H B， Peng J， et al . Environmental Chemistry， 2021， 40（4）： 1193

[22] Xu H， Zhu J， Lei C， et al . Environ Contam Tox， 2016， 97（6）： 770 10.1007/s00128-016-1954-9 27787609

[23] So M K， Miyake Y， Yeung W Y， et al . Chemosphere， 2007， 68（11）： 2085 17368725 10.1016/j.chemosphere.2007.02.008

[24] Huang J H， Wu W， Huang T Y， et al . Environmental Science， 2022， 43（7）： 3562

[25] Zu S Y， Ye F， Motegi M， et al . Environ Sci Technol， 2011， 45（7）： 2887 21384896 10.1021/es103917r

[26] Lam N H， Cho C R， Kannan K， et al . J Hazard Mater， 2017， 323： 116 27106518 10.1016/j.jhazmat.2016.04.010

[27] Hong S， Khim J S， Park J， et al . Sci Total Environ， 2013， 445：136 23333509 10.1016/j.scitotenv.2012.12.040

[28] Nguyen V T， Reinhard M， Karina G Y H . Chemosphere， 2011， 82（9）： 1277 21208640 10.1016/j.chemosphere.2010.12.030

[29] Zhao S .［MS Dissertation］. Beijing： Beijing Jiaotong University， 2022

[30] Paul A G， Scheringer M， Hungerbühler K， et al . J Environ Monit， 2012， 14（2）： 524 22134637 10.1039/c1em10432b

[31] Chen S， Jiao X C， Gai N， et al . Rock and Mineral Analysis， 2015， 34（5）： 579

[32] Huang L Q， Wang W R， Zhang Y T， et al . Environmental Chemistry， 2024， 43（3）： 693

[33] Yang S， Xu F， Wu F， et al . Sci Total Environ， 2014， 470/471： 677 24184545 10.1016/j.scitotenv.2013.09.094

[34] Guo， R， Liu， X， Qiao， X C， et al . Sci Rep-UK， 2020， 10（1）： 4691 10.1038/s41598-020-61651-6 PMC7069980 32170214

